# PSBinder: A Web Service for Predicting Polystyrene Surface-Binding Peptides

**DOI:** 10.1155/2017/5761517

**Published:** 2017-12-27

**Authors:** Ning Li, Juanjuan Kang, Lixu Jiang, Bifang He, Hao Lin, Jian Huang

**Affiliations:** ^1^Center for Informational Biology, University of Electronic Science and Technology of China, Sichuan, China; ^2^Key Laboratory for Neuroinformation of Ministry of Education, Chengdu 611731, China

## Abstract

Polystyrene surface-binding peptides (PSBPs) are useful as affinity tags to build a highly effective ELISA system. However, they are also a quite common type of target-unrelated peptides (TUPs) in the panning of phage-displayed random peptide library. As TUP, PSBP will mislead the analysis of panning results if not identified. Therefore, it is necessary to find a way to quickly and easily foretell if a peptide is likely to be a PSBP or not. In this paper, we describe PSBinder, a predictor based on SVM. To our knowledge, it is the first web server for predicting PSBP. The SVM model was built with the feature of optimized dipeptide composition and 87.02% (MCC = 0.74; AUC = 0.91) of peptides were correctly classified by fivefold cross-validation. PSBinder can be used to exclude highly possible PSBP from biopanning results or to find novel candidates for polystyrene affinity tags. Either way, it is valuable for biotechnology community.

## 1. Introduction

Phage display is a versatile and powerful technology to find ligands for any given target [1–3]. These targets can be a wide variety of substances, such as small molecules, proteins, glycan, cells, organs, and even whole organisms. In traditional phage display experiments, the 96-well plates or microplates are commonly used. Therefore, ligands which bind to polystyrene surface (PS) can appear in the biopanning results unintentionally. On one hand, a high affinity polystyrene surface-binding peptide (PSBP) can help to build a highly effective ELISA system and immobilize proteins or antibodies directly onto the polystyrene plates with minimal conformational changes [4–8]. On the other hand, PSBPs as the target-unrelated peptides (TUPs) are false positive results and may mislead the following experiments [9]. Therefore, it is important to identify if a peptide is likely to be a PSBP in the biopanning results as either the intended peptide or just a TUP.

It is not difficult to identify a PSBP experimentally [9]. However, experimental methods are not economical when dealing with a large quantity of peptides. To save money and time, computational methods for the prediction of PSBP are urgently needed. The machine learning-based approaches have been proved to be quite powerful in dealing with protein and peptide classification problems [10–13]. In this paper, we have proposed a novel PSBP predictor based on support vector machine (SVM) named PSBinder. It can be used to exclude the false positive peptides rapidly and effectively and obtain truly interesting peptides more accurately.

## 2. Materials and Methods

### 2.1. Datasets

We collected the training data from the BDB database released in Jan 2017, which is an information portal to biopanning data [14–16]. The training datasets consisted of the positive and negative datasets. As positive data, the PSBPs were collected from nine different phage display libraries. In order to ensure the comparability between the positive and the negative data, we randomly chose peptides obtained by panning against the same library with targets other than PS. For some libraries that do not have enough number of negative peptides, we collected the peptides in the same length from other libraries as an alternative.

The cysteine amino acids at both ends of the circular peptides were deleted. All peptides harboring ambiguous residues (“B”, “J”, “O”, “U”, “X,” and “Z”) or nonalphabetic characters were excluded. We compared each sequence in the negative dataset with the one in the positive dataset and deleted the identical sequences in negative dataset and replenished the peptides. To exclude possible PSBP crept in the negative data, we used the Generalized Jaccard similarity to keep the peptide sequence similarity of positive and negative data below 90% [17]. Eventually we constructed the negative and positive datasets and each had 104 peptides [4, 18–25]. The whole training dataset is freely available as supplementary online material (available here).

### 2.2. Features and Feature Selection

Extracting the rational features is an extremely significant step in constructing a well-behaved prediction model [26, 27]. Several kinds of typical features, such as single amino acid compositions (AACs) and dipeptide compositions (DPCs), amino acid physicochemical properties, and the pseudo-amino-acid composition, are widely used in developing classifiers for protein and peptide prediction. The classifiers based on these features have shown excellent performance [10, 28–32].

It is a wise method to count the amino acid frequencies of protein sequences to express the feature of protein sequences. We can distinguish different types of protein through the difference in the frequency distribution of amino acids between sequences. And this is also applicable for peptide sequences; we chose the AACs as the feature. In order to compensate for the lack of intrinsic link of the amino acid, we also import the DPCs. A peptide sequence can be composed of 20 amino acids (ACDEFGHIKLMNPQRSTVWY) at random in each position, so a peptide that contains *L* amino acids could be expressed as

(1) $$\begin{array}{c} \beta =\left(\;\beta_{1},\beta_{2},\ldots ,\beta_{L}\;\right). \end{array}$$

*β*_1_, *β*_2_, and *β*_*L*_ represent the first, the second, and the *L*th amino acid of the peptide sequence *β*. And the definition of AAC and DPC is as follows:

(2) AACi=xi∑i=120xiDPCj=yj∑j=1400yj,

where *i* stands for one of the 20 amino acids and *j* one of the 400 dipeptides. *x*_*i*_ denotes the number of residues of each type and *y*_*j*_ represents the number of dipeptides of each type in each sequence.

In order to build a prediction model with high efficiency, AAC and DPC were further screened to drop the irrelevant, redundant, and noisy features through fselect.py script supported by LIBSVM3.22 [33]. Feature selection was performed as follows. The feature was put into an initially null set in descending order by accuracy one by one and the accuracy of each set was calculated when an element was added in. When the prediction accuracy reached the highest value, we chose the set as the optimal feature subset. After the above procedures, we finally acquired the optimized AAC (OAAC) and the optimized DPC (ODPC).

### 2.3. Support Vector Machine

In machine learning methods, the support vector machine is a supervised learning model algorithm for regression analysis and prediction of data. The SVM has gained increasing popularity and also been extensively used in the field of bioinformatics [34–37]. We applied SVM to the analysis and prediction of PSBP. The SVM model was developed by using LIBSVM3.22 [33], which is an integrated software for support vector classification. The best error factor **c** and the kernel function variance **g** needed to build the model can be found by the software's built-in python script grid.py. In order to visualize the prediction results, the parameter *b* is set to 1 in the process of model training.

### 2.4. Prediction Assessment

N-fold cross-validation is often used to evaluate the predictive performance of statistical predictive models. The advantage of the N-fold cross-validation method is the simultaneous and repetitive use of randomly generated subsamples for training and verification. In this work, all established models were evaluated by using fivefold cross-validation, where the entire dataset was randomly divided into five groups, each containing an equal number of peptides. Four groups were used for training and the remaining one was used for testing. This process would be repeated five times. In such a way, each group was used as the test group once. Eventually the average prediction accuracy of five kinds of combination was calculated as the final accuracy of one model.

To evaluate the performance of the prediction models, we used four indicators: sensitivity (Sn), specificity (Sp), accuracy (Acc), and Matthews correlation coefficient (MCC).

(3) Sn=TPTP+FN,Sp=TNTN+FP,Acc=TP+TNTP+FN+FP+TN,MCC=TP×TN−FP×FNTP+FPTP+FNTN+FPTN+FN.

In the above formulas, TP and TN represent the number of correctly predicted PSBPs and non-PSBPs, respectively and FP and FN represent the number of wrongly predicted PSBPs and non-PSBPs, respectively. MCC is one of the most robust parameters in any class predictive approach. A MCC equal to 1 is deemed to be the best prediction, whereas 0 is for a completely random prediction and −1 is an absolutely adverse prediction. In addition, the competence of the model is illustrated with the Receiver Operating Characteristic (ROC) curve. The area under the ROC curve (AUC) is used as the performance measure. For a perfect prediction, the maximum value of the AUC equals 1.0. For a random guess, the AUC equals 0.5.

### 2.5. Online Web Service

We used Perl to write the common gateway interface script for the web service. The feature extraction script was written by Python. The web service allows user to submit peptide sequences in FASTA format or as plain text. The result will be returned and displayed in a table after prediction.

## 3. Results

### 3.1. The Establishment of Prediction Model and Performance Evaluation

In this study, the positive dataset contains 104 peptide sequences, and the negative dataset is composed of 104 peptide sequences with the same length and almost the same source to the corresponding positive peptides. According to formula (2), each sequence of 420 features can be calculated. By filtering these redundant and high dimensional features, we finally obtained 9 OAAC and 146 ODPC. The model built with ODPC attains the maximum accuracy of 87.02% and an impressive MCC of about 0.74 (Table 1). These indicators show the excellent performance and strong generalization ability of the predictor.

**Figure 1:**
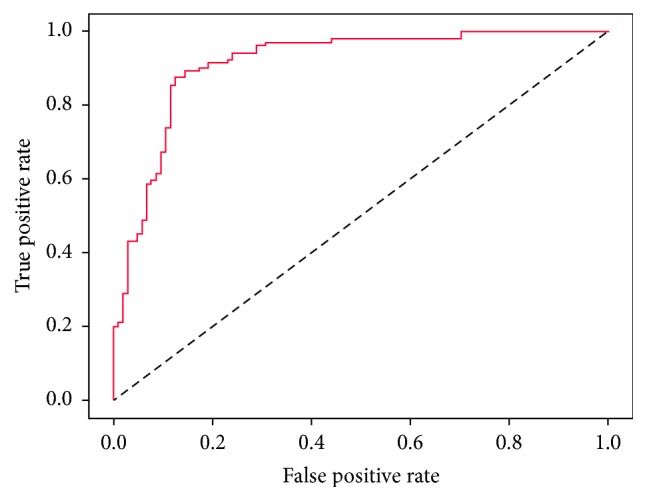
The ROC curve graph of the prediction model based on ODPC.

To more intuitively illustrate the efficiency of the predictor, we also used the ROC curve to graphically describe the performance of the predictor.  Figure 1 is the ROC curve of the predictor constructed by the ODPC. The abscissa of the graph represents the false positive rate of the prediction model and the ordinate of the graph represents the true positive rate. In a rational situation, we expect a true positive rate equal to 1 and false positive rate equal to 0 and at this time the AUC is 1. The AUC area of our predictor is as high as 0.91, which demonstrates that the predictive performance of our predictor is pretty good.

### 3.2. Comparison with Other Machine Learning Methods

In order to prove that the prediction model based on SVM is better than the prediction model based on other machine learning methods, we used the ODPC to build predictive models based on Naive Bayes, Logistic Function, Random Forest, LibD3C [38], and Decision Tree J48, respectively, [39]. As the fivefold cross-validation results shown in Table 2, the average accuracy of the SVM model is approximately 3.82%, 5.95%, 9.12%, 11.06%, and 25.97% higher than that of Naive Bayes, Logistic Function, Random Forest, LibD3C, and Decision Tree J48 classifiers, respectively. This indicates a better performance of our SVM-based model.

### 3.3. Online Web Service

In order to facilitate its usage among relevant researchers, we integrated this tool with SAROTUP, which has been developed into a suite of web tools for identifying or predicting target-unrelated peptides. Users can directly access the PSBinder and get results at http://i.uestc.edu.cn/sarotup/cgi-bin/PSBinder.pl.

## 4. Discussion

In the published papers, the PS-binding motifs such as WXXW [19], FHXXW [21], and WXXWXXXW [23] had been found in many PSBPs. However, there are many PSBPs that do not have the typical motifs [23]. There are no tools capable of rationally predicting PSBP when peptides bear no such motifs. PSBinder was modeled by the dipeptide features, which successfully responds to these situations.

Our model was built with 146 features. The top three features are WG, WF, and WE. According to the analysis of amino acid composition, we found that the most frequently occurring amino acids were W, Y, and F. It indicates that the hydrophobic amino acids with the benzene ring may play an important role in binding polystyrene. And all the hydrophobic amino acids appear in our features. Thus, when a peptide has the amino acids with the benzene ring and is accompanied by many hydrophobic amino acids, it may be a PSBP.

In addition, after the completion of our predictor, a paper published very recently reported a PSBP with the sequence of VHWDFRQWWQPS [40]. As the paper reported, this sequence does not have typical PS-binding motifs. Since this peptide is not seen in the training datasets, we used it as an independent case test. PSBinder predicted this peptide as a PSBP (the probability is about 0.88), which agreed with the experimental result.

## 5. Conclusions

In this paper, we developed a predictor based on SVM to detect if a peptide is a PSBP. The model constructed by optimized dipeptide features had a good performance. The maximum accuracy of 87.02% was achieved with 0.74 MCC, 88.46% sensitivity, and 85.58% specificity, respectively. In addition, in order to facilitate its usage, the SVM-based model was implemented into an online web service called PSBinder. It is practical and freely available at http://i.uestc.edu.cn/sarotup/cgi-bin/PSBinder.pl. PSBinder would be a useful tool to predict PSBPs, whether as TUPs or intended peptides. It will help to speed up the experiment process and facilitate the development of biological products.

## Figures and Tables

**Table 1:** Performances of SVM-based models trained with different features.

Feature	Sn (%)	Sp (%)	Acc (%)	MCC
Optimized amino acid composition (OAAC)	66.35	79.81	73.08	0.47
Optimized dipeptide composition (ODPC)	88.46	85.58	87.02	0.74

**Table 2:** The prediction performances of various machine learning methods.

Machine learning methods	Sn (%)	Sp (%)	Acc (%)	MCC
*Support vector machine*	*88.46*	*85.58*	*87.02*	*0.74*
Naive Bayes	83.70	82.70	83.20	0.66
Logistic Function	76.90	86.50	81.70	0.64
Random Forest	73.10	82.70	77.90	0.56
LibD3C	78.72	73.68	75.96	0.52
Decision Tree J48	48.10	74.00	61.05	0.23

## Supplementary Materials

Supplementary Materials The supplementary dataset consists of the positive and negative datasets. Each dataset contains 104 peptides which were collected from the BDB database [16]. The “positive.txt” is the positive dataset; the “negative.txt” is the negative dataset.
